# Changes in Milk Protein Functionality at Low Temperatures and Rennet Concentrations

**DOI:** 10.3390/foods13030447

**Published:** 2024-01-30

**Authors:** Mahmoud E. A. Hamouda, Prafulla Salunke

**Affiliations:** Dairy and Food Science Department, Midwest Dairy Foods and Research Center, South Dakota State University, Brookings, SD 57007, USA; mahmoud.hamouda@sdstate.edu

**Keywords:** rennet, skim milk, low temperature, milk protein functionality

## Abstract

This study aimed to evaluate the influence of low-concentration rennet on the chemical, rheological characteristics, and protein fractions of skim milk (SM) at 4 ± 1 °C. Skimmed milk (SM) was divided into four lots of 500 mL, and diluted rennet (1:10,000) was added at different levels at 4 ± 1 °C. The treatments included control (no rennet), T1 (0.001 mL/rennet), T2 (0.01 mL rennet), and T3 (0.1 mL rennet) treatments, which were incubated for 24 h. The sampling was performed at 0, 1, 2, 6, 12, and 24 h, and the SM after incubation time was heated to 73 °C/16 s to denature the rennet enzyme. Skim milk samples (SMS) (control and rennet-added samples) were evaluated for proximate composition, capillary gel electrophoresis (CGE), hydrodynamic diameter, zeta potential, and rheology at 0, 1, 2, 6, 12, and 24 h. Foaming ability, foaming stability, water-holding capacity (WHC), oil emulsifying activity (OEA), and emulsion stability (ES) were performed at 0, 12, and 24 h of incubation time. There was a significant (*p* < 0.05) increase in non-proteins by 0.50% and in non-casein nitrogen by 0.81% as incubation progressed. The results showed that aggregation or curd was not formed during storage time. The CGE data indicated that increasing the rennet concentration had a significant (*p* < 0.05) effect on decreasing κ-CN, and breakdown increased at higher levels of rennet usage. There was a significant (*p* < 0.05) increase in the hydrodynamic diameter and a decrease in the zeta potential values in rennet-added samples at the end of the incubation time (24 h). The rheological results showed no changes in the storage modulus (G′), loss modulus (G″), or viscosity values. Increasing the rennet amount and storage time led to a significant (*p* < 0.05) decrease in the foaming ability and foaming stability and a significant (*p* < 0.05) increase in the oil emulsifying activity and emulsion stability of rennet-added SMS. This study concluded that milk protein functionality can be changed without aggregating or curd formation, and rennet milk can be processed.

## 1. Introduction

Dairy ingredients are versatile and used in various foods for their nutritional values and critical functional properties. Among the various milk constituents, milk proteins are prominent. It is widely recognized that milk protein is a powerful nutrient with many health and wellness benefits. Milk proteins are also essential for all dairy products’ structural and textural networks and play a critical role in their functional properties, including texture, water binding, water-holding capacity (WHC), viscosity, gelation, emulsifying, and foaming [1,2,3,4]. Milk protein products are the first ideal protein choice for the dairy and food industries when developing high-protein foods with certain functional properties. Protein-based foods and beverages, including those naturally high in protein, like yogurt and fortified foods, continue to gain popularity. The major milk protein fraction comprises caseins (~80%) and whey or serum proteins (~20%), each having different functions and characteristics. Caseins are the most essential and valuable component of milk from a product and technological perspective [5], and most of the functional properties of the casein micelle depend on its surface properties [5,6] and molecular structure [7]. Hence, the source and treatment of milk protein as an ingredient become essential for functionality.

Casein-rich products can be produced using different techniques, such as acidification (acid-coagulated cheeses, acid casein powder), enzyme hydrolysis using chymosin (natural cheeses, rennet casein powder), condensing (concentrated milk), spray drying (nonfat dry milk, skimmed milk powder), or membrane filtration techniques (milk protein concentrate (MPC) or micellar casein concentrate (MCC)). Since high-protein powders have poor solubility, some water-soluble forms of casein are made by adding alkali, such as sodium hydroxide, calcium hydroxide, etc. [8]. Each approach gives casein unique properties and different functionalities when used as an ingredient in various product formulations. For example, when used, chymosin produces casein, which is a native casein (para casein) that is highly insoluble, and its κ-casein portion is devoid of glycomacropeptide (GMP). Acid and rennet casein yield casein in destabilized as well as insoluble forms. In many products, rennet casein powder is preferred as it provides 100% intact casein and maximum viscosity per gram of protein. Even though it is insoluble in water, rennet casein powder is the preferred ingredient in many product applications where functionality is extracted in the presence of emulsifying salts. Meanwhile, casein from membrane processes is a soluble protein in micellar form [9,10].

Nowadays, membrane separation is used for large-scale fractionation or the separation of individual components and is considered economical, efficient, and environmentally friendly. This has led to the development of well-defined fractions, which help us use or extract benefits optimally, such as their functional properties and health benefits, in diverse applications. MPC and MCC manufacture involve separation through physical means to produce a product. The proteins in these products are in their native state, i.e., the casein remains in a form that resembles the original casein micelles in milk and has κ-CN and GMP portions of κ-CN in their intact and native forms. The GMP portion of κ-CN is rich in carbohydrates and provides a negative charge on the surface of the casein micelle. This unique property provides steric stability to the casein micelle via electrostatic repulsion of adjacent micelles [5,6]. This GMP portion with a negative charge interferes with the formation of the protein-based network and impacts the functionality and performance of these products. When high levels of MPC or MCC are used, the problems in functional performance may be related to the κ-CN present in the native casein micelle [10]. Hence, MPC and MCC alone cannot deliver the critical functional properties required in products such as high heat stability, emulsions, yogurt, and processed cheese products. The functionality of the milk proteins has been improved by modifying the processing conditions, such as pH and temperature, or by combining or using chymosin or crosslinking enzymes, such as Transglutaminase [10,11].

Changing the functionality of milk proteins is important in dairy science, as it directly influences the quality and variety of dairy products [12]. Changes in casein structure by processes like heat treatment, fermentation, or enzymatic action (such as with rennet) can enhance its characteristics during dairy processing [13]. For instance, modifying the structure of caseins affects cheese texture. Additionally, these changes can be used to produce new dairy products. Therefore, understanding and controlling these changes is essential for producing a wide range of dairy products with desired properties, making it a significant focus in dairy processing and product development.

Over the years, technologies have been developed to produce various products by re-solubilizing milk proteins using emulsifying salts when these ingredients are used in the formulation. Also, several studies were carried out to change the functionality of milk proteins without aggregate or curd formation, a process typically associated with cheese manufacturing. These studies have focused on modifying milk protein structure and interactions under controlled conditions, using different techniques like controlled enzymatic hydrolysis [14], crosslinking using transglutaminase [10,11], and high-pressure processing [15]. The protease enzymatic treatment under controlled conditions of low temperatures has been used in acid whey to enhance whey proteins’ functional properties and solubility for use in beverages and emulsions [16]. Similarly, high-pressure treatment has been reported to change food protein fractionations and improve their functional properties like foaming and gelling [17]. These methods can diversify dairy product formulations and extend the applications of milk proteins beyond traditional cheese manufacturing.

Milk-clotting enzymes belong to aspartic proteinase (EC 3.4.23), and chymosin (EC 3.4.23.4) or rennet enzyme is one of them [18]. The chymosin enzyme hydrolyzes the Phe105-Met106 bond of κ-CN on the casein micelle surface, destabilizing the casein micelles and aggregating various destabilized micelles in the presence of calcium. This leads to the formation of curd and the expulsion of whey. The chymosin action drastically changes the casein micelle surface, including the removal of κ-CN hairs, and the change in negative charge leads to significant changes in functionality. Fundamentally, rennet action removes a GMP from the casein micelle and changes the structure and stability of the casein micelle, leading to curd formation [10]. Rennet, like other enzymes, has an optimum temperature for its activity. The optimum temperature for rennet activity in milk is around 35 °C to 40 °C [19]. At this range of temperatures, the enzyme chymosin is most effective at milk coagulating and provides ideal conditions for the enzyme to act on κ-CN, and when more than 85% κ-CN is hydrolyzed, it leads to coagulation or curd formation. The chymosin-induced coagulation of bovine milk does not occur below about 18 °C [20,21,22,23,24] due to the influence of temperature on the secondary (nonenzymatic) phase [25]. The visually observable aggregation and gelation essentially do not occur below ~18 °C; however, it has been reported that casein micelles in milk do aggregate at 10 °C, but at a slow rate, and beta-casein, likely through its hydrolysis, plays a role in the aggregation of chymosin-altered casein micelles at low temperatures [26]. This phenomenon can be used to produce new ingredients with altered or tailored functionality without coagulating the milk.

Functional properties of proteins may be altered using other proteases, depending on the nature of the protein substrate, the type of enzyme used, and the extent of hydrolysis [27,28]. The hydrolyzed proteins tend to be characterized by a bitter taste and some off flavors, which limit their use in the food industry. However, cheese or rennet casein powder made using chymosin has traditionally been a primary source of casein or intact casein. Rennet is used for the manufacture of cheese or rennet casein powder [29] by removing GMP as well as the negative charge on the casein micelle and changing the functionality of the products. This rennet action makes milk proteins in cheese or rennet casein powder insoluble and extremely difficult to process as a liquid. The main reason for the changed functionality observed in natural cheese and rennet casein powder compared to native protein products such as nonfat dry milk (NDM), milk protein concentrate (MPC), or micellar protein concentrate (MCC) is because of the surface modification of casein micelles. If the controlled rennet action to modify the milk proteins is carried out without making the milk proteins insoluble, new products can be developed with altered functionality. Our study hypothesized that treating skim milk with a low amount of chymosin (rennet) under controlled low-temperature conditions of 4 °C can change the functional properties of milk proteins without leading to curd formation. This approach aims to take advantage of rennet’s highly specific enzymatic action on milk proteins to modify their structure and functionality while avoiding curd formation. This could have significant implications for the manufacturing of novel dairy products. Hence, the experiment was planned to study the changes in milk protein functionality at low temperatures and rennet concentrations.

## 2. Materials and Methods

### 2.1. Experimental Design

Pasteurized skim milk obtained (Dairy Plant, South Dakota State University, Brookings, SD, USA) and divided into four lots of 500 mL each. Diluted rennet (mL/100 mL) was added at different levels at 4 ± 1 °C, as shown in Table 1, and incubated for 24 h. Rennet (CHY-MAX Extra, ~800 international milk coagulating units (IMCU mL^−1^) was obtained from Chr Hansen, Milwaukee, WI. The rennet was diluted (1:10,000) times before being utilized. The sampling was performed at 0, 1, 2, 6, 12, and 24 h. After incubation, the control and rennet-added samples were heated to 73 °C/16 s to denature the rennet enzyme. This experiment was repeated three times using three different batches of skim milk. Skim milk samples (SMS control and rennet-added samples) were analyzed for proximate composition, rheological characteristics, capillary gel electrophoresis (CGE), hydrodynamic diameter, zeta potential, and functional properties (foam properties, WHC, oil emulsifying activity, and emulsion stability).

### 2.2. Proximate Composition and pH

The proximate composition of initial skim milk was performed using Lactoscan spectroscopy technology (Bently Instruments, Inc., MN, USA). AOAC [30] methods were used to determine total nitrogen (AOAC, 2000 method 991.20; 33.2.11), non-protein nitrogen (AOAC, 2000 method 991.21; 33.2.12) and non-casein nitrogen (AOAC, 2000 method 998.05; 33.2.64) in SMS using the Kjeldahl method [30]. The total protein, cfv NCN, and NPN protein were calculated as nitrogen and multiplied by 6.38. Proximate analyses were performed at 0, 1, 2, 6, 12, and 24 h. A digital pH meter (Hanna Instruments, Inc., Woonsocket, RI, USA) was used for the determination of the pH of SMS (control and rennet-added samples), as described by the method given in (AOAC, 2000 method 973.41) [30].

### 2.3. Capillary Gel Electrophoresis (CGE) 

Various protein fractions in the SMS (control and rennet-added samples) were determined using CGE [31]. For this analysis, samples were first diluted to achieve a protein concentration of around 1% using distilled water. Subsequently, a volume of 10 µL from each diluted sample was mixed with 85 µL of a sample buffer (ProteomeLabTM SDS-MW Analyses Kit, Beckman-Coulter, Fullerton, CA, USA), and 5 µL of β-mercaptoethanol was added into a PCR-vial (Fisher Scientific Co LLC., Florence, KY, USA) and heated in a water bath at 90 °C for 15 min. The samples were analyzed with the CGE (Beckman P/ACE MIDQ, Beckman-Coulter, Fullerton, CO, USA), which was equipped with a UV detector set at 214 nm. The test was performed using a 50 µm bare fused silica capillary (20.2 cm effective length from the inlet to the detection window). A capillary preconditioning method (basic rinse: 0.1N NaOH, 5 min, 50 psi; acidic rinse: 0.1N HCl, 2 min, 50 psi; distilled water rinse: 2 min, 50 psi; and SDS gel rinse: 10 min, 40 psi) was run every three samples. Then, the sample was electrokinetically introduced at 5 kV for 20 s. The separation occurred using the following conditions: a constant voltage of 15 kV, a temperature of 25 °C, and 20 bar pressure with reverse polarity in the SDS molecular weight gel buffer. Molecular weight standards (ProteomeLab and Beckman-Coulter) and available pure milk protein fractions (Sigma-Aldrich, Inc., St. Louis, MO, USA) were also separated using the above-mentioned method to calculate migration times. The area of each identified peak was calculated from the electropherogram using a valley-to-valley approach, according to [31,32]. The area of each was identified as individual CN fraction (αS1, αS2, β, κ, and γ-CN), SP fractions (α-LA, β-LG), and peptides (peaks between 10 and 20 kDa) were calculated as a percentage of the total area (positive peaks). The quantification involves calculating the area of each casein (CN) fraction (αS1, αS2, β, κ, and γ-CN) and serum protein (SP) fractions (α-LA, β-LG) as well as peptides (with molecular weights between 10 and 20 kDa). This was expressed as a percentage of the total area, considering only the positive peaks.

The κ-CN breakdown rate % at 12 and 24 h was calculated using the following equation:

(1) $$\mathrm{The}\;\kappa -\mathrm{CN}\;\mathrm{breakdown}\;\mathrm{rate}\;\%\;\mathrm{at}\;12\;h=\frac{\kappa -\mathrm{CN}\%\;\mathrm{at}\;zero\;time-\kappa -\mathrm{CN}\%\;at\;12\;h}{\kappa -CN\%\;at\;zero\;time}\times 100$$

(2) $$\mathrm{The}\;\kappa -\mathrm{CN}\;\mathrm{breakdown}\;\mathrm{rate}\;\%\;\mathrm{at}\;24\;h=\frac{\kappa -\mathrm{CN}\%\;at\;zero\;time-\kappa -CN\%\;at\;24\;h}{\kappa -\mathrm{CN}\%\;at\;zero\;time}\times 100$$

### 2.4. Hydrodynamic Diameter and Zeta Potential

The mean values of hydrodynamic diameter (µm) and zeta potential (mv) of protein in SMS (control and rennet-added samples) were measured using a particle size analyzer (Litesizer 500, Anton Paar, Ashland, VA, USA) at 27 °C, according to a method described by Olson et al. [33]. One ml of control and rennet-added samples were diluted using distilled water 1:1000 times to reach transmittance above 85%. The cuvette (Omega, Anton Paar, USA), designed to determine particle size and zeta potential, was filled with a diluted sample. The hydrodynamic diameter (µm) and zeta potential (mv) values were measured in triplicate.

### 2.5. Rheological Characteristics

The rheological characteristics of the SMS (control and rennet-added samples) were performed using an Anton Paar rheometer (MCR 92, Anton Paar, Graz, Austria) equipped with a cylinder cup (42.01 mm inner diameter) and bob (38.69 mm outer diameter, 60.02 mm length effect, 143.8 mm active length, and 72.50 mm positioning length) [34]. The temperature of samples (60 mL) was maintained at 25 °C before performing the test. The storage modulus (G′), which indicates the elastic or ‘solid-like’ behavior of a material, and the loss modulus (G″), which depicts the viscous or ‘liquid-like’ behavior of a material, were measured at 25 °C at an applied shear strain of 0.5% and an angular frequency ranging from 0 to 115 rad/s. Data points were collected at a rate of 5 rad/s. Viscosity (mPa·s) of control and rennet-added samples was measured using the same equipment at a share stress of 1 to 1000 (1/s) at a 10 shear rate per 3 s.

### 2.6. Foam Properties

Foamability and the foaming stability of SMS (control and rennet-added samples) were measured using the method previously described by Petkova et al. [35]. Thus, 200 mL of each sample was placed in a glass bowl at room temperature (25 °C). The height of skim milk (H0) was determined before homogenizing, using a hand blender (Vavsea, Alhambra, CA, USA) at 3000 rpm for 3 min. After blending immediately, the foam height (H2) and the total height (H1) were estimated. Subsequently, SMS was left for 10 min to rest; then, the new foam heights (H3) were determined. Foamability and foaming stability were calculated using the following equations:

Foamability (%) = (H1 − H0)/H0 × 100(3)

Foaming stability (%) = H3/H2 × 100(4)

### 2.7. Determination of Water-Holding Capacity (WHC)

The water-holding capacity was determined as described by Li et al. [36] with some modifications. SMS (control and rennet-added samples) were incubated at 30 °C for 15 min in a water bath. A total of 10 mL of the sample was transferred into a 10 mL graduated centrifuge tube. Subsequently, centrifugation was performed using a Sorvall ST plus series centrifuge (ThermoFisher Scientific, Karlsruhe, Germany) at a force of 2000× *g* at 30 °C for 15 min. After centrifugation, the water-holding capacity was calculated as the percentage ratio of the sediment volume (in mL) to the initial sample volume (in mL).

### 2.8. Oil Emulsifying Activity and Emulsion Stability 

The oil emulsifying activity and emulsion stability were evaluated according to the method described by Benvenutti et al. [37], with some modifications. SMS (control and rennet-added samples) and sunflower oil (Hy-Vee, Brookings, SD, USA) were incubated in a water bath maintained at 30 °C for 15 min. Then, 70 mL of SMS was mixed with 30 mL of sunflower oil. Subsequently, milk and oil were mixed using a lab-scale high sheer homogenizer (Polytron^®^pt 2500 E, Kinematica AG, Malters, Switzerland) at 15,000 rpm for 2 min at 30 °C. After homogenization, 10 mL of the mixture was transferred in the 10 mL volumetric graduated centrifuge tube and centrifuged at 30 °C for 15 min at a force of 2000× *g* using the Sorvall ST plus series centrifuge (ThermoFisher Scientific, Karlsruhe, Germany). The emulsion volume was recorded, and the oil emulsion activity was calculated as shown in Equation (5).

(5) $$\mathrm{Emulsion}\;\mathrm{activity}\;\%=\frac{Emulsion\;volume\;(in\;mL)}{initial\;sample\;volume\;(in\;mL)}\times 100$$

To determine emulsion stability, the same tube samples were pasteurized in a hot water bath at 80 °C for 30 min, followed by cooling at 30 °C for 15 min. The tubes were recentrifuged at 30 °C for 15 min at a force of 2000× *g* using the Sorvall ST plus series centrifuge (ThermoFisher Scientific, Karlsruhe, Germany), and the emulsion volume was recorded. The emulsion stability was calculated as in Equation (6).

(6) $$\mathrm{Emulsion}\;\mathrm{stability}\;\%=\frac{Emulsion\;volume\;after\;recentrifuging\;(in\;mL)}{initial\;sample\;volume\;(in\;mL)}\times 100$$

### 2.9. Statistical Analyses

Data were analyzed using Costat 6.303 software (Cohort software, Monterey, CA, USA) to determine the differences among the means of the treatment samples. Statistical analysis was also performed to study the effects of treatment (rennet amount) and storage time at 4 °C and the interaction between them. An ANOVA was performed to obtain the mean squares and *p*-values, using the two-way procedure completely randomized design available in Costat 6.303 software. When a significant treatment, time, or interaction effect (*p* < 0.05) was detected, the differences among means were compared using the least significant difference test.

## 3. Results and Discussion

### 3.1. Proximate Composition

The average proximate composition of initial skim milk is listed in Table 2. Skim milk had a low fat content of 0.18% with a pH of 6.62. The lactose content was 4.93%, while the total solids (TS) and solids not fat (SNF) were 9.72% and 9.54%, respectively. The skim milk had 3.61% total protein (TP), 0.67% non-casein nitrogen (NCN), and 0.18% non-protein nitrogen (NPN). These results indicate a typical composition and normal protein distribution of skim milk [38].

Table 3 presents the statistical analysis of the effects of rennet amount (treatments), different incubation periods (time), and the interaction between them (treatments × time) on pH, TP, NCN, and NPN values of SMS (control and rennet-added samples) at 4 °C. Treatments and time significantly (*p* < 0.05) affected pH, NCN, and NPN, but they did not affect TP. The interaction between treatments and time also showed significant (*p* < 0.05) effects on NCN and NPN; however, it did not have a significant impact (*p* < 0.05) on the pH and TP.

The chemical composition of SMS (control and rennet-added samples) over 24 at 4 °C is presented in Table 4. After zero hours, the obtained results showed that all SMS (control and rennet-added samples) revealed a typical skim milk composition, with pH (6.63), TP content (3.53%), NCN (0.67%), and NPN (0.18%). Over 24 h at 4 °C, compared to the control, the pH values of rennet-added SMS showed a significant (*p* < 0.05) decrease. This rennet enzymatic action is a common reason for changes in the pH levels since the protein breakdown process releases acidic amino acids, subsequently lowering the pH levels [39]. Moreover, some microorganisms during incubation can break down minimal amounts of lactose into lactic acid, which is responsible for decreasing the pH of milk [40].

The TP values of SMS did not show a significant (*p* > 0.05) effect, and they were maintained at around 3.58% to 3.60%. This could be because the total protein content contains all protein fractions and nitrogenous compounds in milk, even peptides that accumulate from the enzymatic breakdown of milk proteins [41]. However, a significant increase (*p* < 0.05) in NCN and NPN values was observed with higher rennet concentrations and as storage time advanced, particularly in the T3 after 24 h, which recorded 1.48% of NCN and 0.68% of NPN. These results suggest a gradual breakdown of casein into smaller peptides and free amino acids, induced by rennet enzymatic action, which became apparent at higher concentrations and extended incubation times [42].

### 3.2. Capillary Gel Electrophoresis (CGE)

CGE is a highly efficient analytical technique used to fractionate, separate, and analyze milk proteins based on their molecular weight (MW) and charge [43]. This process allows for the precise quantification and characterization of individual protein fractions in milk. CGE is particularly useful for detecting changes in milk protein composition due to processing. Among the casein (CN) fractions, the β-casein (β-CN) exhibited earlier migration compared to αS1-casein (αS1-CN) despite αS1-CN having a lower molecular weight. Similar results were obtained by [44,45]. The electrophoretic velocity of αS1-casein (αS1-CN) is reduced due to its regions being rich in negative charge, which, in SDS, causes an extension of its conformation. This extended conformation increases apparent molecular size, resulting in a slower migration rate during SDS-polyacrylamide gel electrophoresis (SDS-PAGE) conditions [31,44,46]. κ-CN has a low molecular weight (MW) compared with other caseins. However, it appeared last after all other CN fractions, and this late migration of κ-CN is attributable to the glycosylation of κ-CN [31]. Alterations in protein fractions, like enzymatic hydrolysis or crosslinking, can change MW, resulting in variations in peak heights and migration times during analysis.

Statistical analysis of the effects of rennet amount (treatments), different incubation periods (time), and the interaction between them (treatments × time) on different protein fractions of SMS (control and rennet-added samples) at 4 °C is shown in Table 5. The rennet amount (treatments) significantly (*p* < 0.05) affected LMWP, γ-CN, β-CN, κ-CN, and HMWP but did not have a significant (*p* > 0.05) effect on αS1-CN, αS2-CN, α-LA, and β-LG. Different incubation periods (time) also had a significant (*p* < 0.05) impact on LMWP, α-LA, β-LG, γ-CN, αS1-CN, κ-CN, and HMWP and a non-significant (*p* > 0.05) impact on β-CN and αS2-CN. The interaction (treatments × time) showed a significant (*p* < 0.05) impact on LMWP, γ-CN, κ-CN, and HMWP; however, it did not have a significant impact (*p* < 0.05) on the other protein fractions.

**Figure 1:**
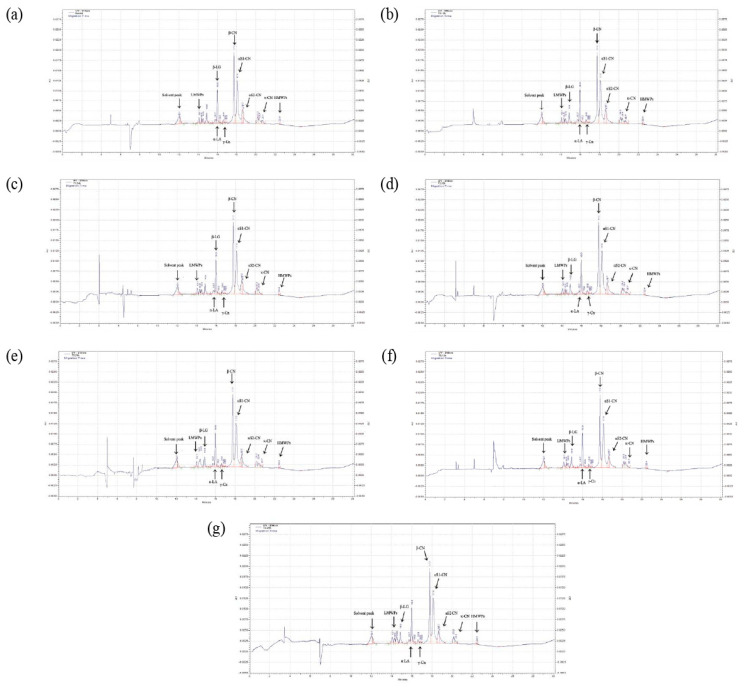
Capillary gel electrophoretogram of skim milk sample include the following: (**a**) control during 24 h of incubation; T1, T2, and T3 during at 0, 1, 2, and 6 h of incubation; (**b**) T1 at 12 h of incubation; (**c**) T1 at 24 h of incubation; (**d**) T2 at 12 h of incubation, (**e**) T2 at 24 h of incubation; (**f**) T3 at 12 h of incubation; and (**g**) T3 at 24 h of incubation. AU = absorbance units.

The different protein fractions observed and determined in the CGE electropherogram of SMS (control and rennet-added samples) are shown in Table 6. Rennet acts on κ-CN in milk, splitting it at a specific point between the Phe105 and Met106 amino acids. This split or hydrolysis weakens the structure of the casein micelles, making them less soluble and causing the casein proteins to clump together, which leads to a change in their MW and, hence, a change in their volume peaks, increasing or introducing new peaks of lower MW. Although rennet addition and rennet-added SMS (T1, T2, and T3) did not show remarkable changes in all protein fractions (CN or SP), migration at 0, 1, 2, and 6 h and peaks were approximately similar in all treatments (Figure 1a). The percentage of peak areas of (CN, SP, and LMWP) fractions in SMS (control and rennet-added samples) after 6 h of storage ranged from 27.73 to 29.03% for β-CN, 33.53 to 35.49% for αS1-CN, 8.77 to 9.59% for αS2-CN, 7.59 to 8.47% for κ-CN, 1.31 to 1.49% for γ-CN, 4.56 to 5.25% for α-LA, 10.93 to 11.30% for β-LG, and 2.65 to 2.82% for LMWP. It has been reported that the mean CN fractions in normal milk, including β-CN, αS1-CN, αS2-CN, κ-CN, and γ-CN, were approximately 33.8, 34.4, 8.5, 8.5, and 3.0%, respectively, and the means of SP fractions of β-LG and α-LA were 9.5 and 5.1%, respectively [22,47,48], which are similar to our results. Also, at the same time (6 h), the average CN/TP ratio in all SMS ranged from 80.65 to 81.53%, which was similar to the value of 82.33% determined in skim milk [31]. These results reflect that adding rennet with different concentrations did not affect the protein fractions of rennet-added SMS until 6 h of incubation at 4 °C. The incubation temperature at 4 °C was substantially below the optimal temperature range for chymosin, which ranged from 35 °C to 40 °C [49]. Consequently, chymosin exhibits markedly reduced activity, which decreases efficacy in milk protein breakdown processes [50].

As the incubation time progressed (12 and 24 h at 4 °C), rennet-added SMS showed a highly significant (*p* < 0.05) decrease in κ-CN and a gradual increase (*p* < 0.05) in LMWP, which indicated that a higher concentration of rennet at extended incubation time (12 and 24 h) led to more κ-CN breakdown into para-κ-CN and GMP (Figure 1b–g). Para-κ-CN fragments have low MW compared to milk protein fractions. Due to their reduced LMWP and altered functional properties post-cleavage, they behave more like a low-molecular-weight peptide component. Thus, it appears in the category of LMWP in CGE. The hydrophilic C-terminal end of the κ-CN molecule, after breaking down by chymosin, is known as the GMP (carbohydrate part), and it is difficult to detect using UV detection (214 nm) in CGE [51].

The unequal rates of decrease in κ-CN and rates of increase in LMWP peptides can be potentially attributed to the limitations of CGE in detecting these peptides (GMP). Some peptides may have been identified as γ-CN or aggregated to form higher-molecular-weight peptides. This could explain the significant increase (*p* < 0.05) observed in γ-CN and high-molecular-weight peptides as the rennet concentrations increased over time and there was an interaction between them (Table 6), which could be actual remnants of κ-CN resulting from enzymatic cleavage.

**Figure 2:**
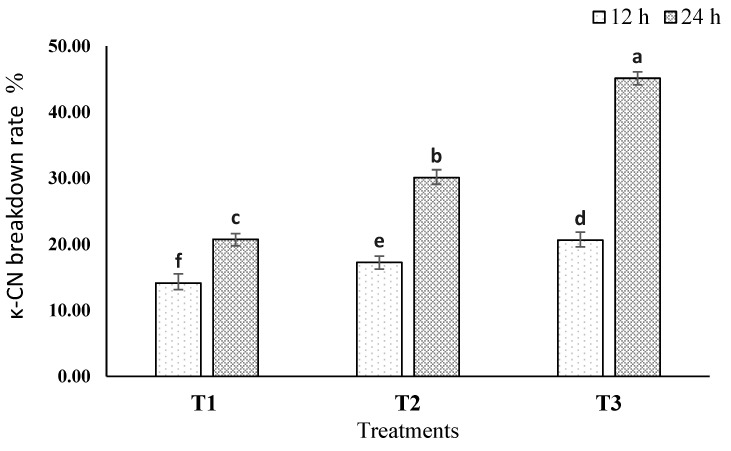
κ-CN breakdown rate % of skim milk sample (T1, T2, and T3) at 12 and 24 h of incubation. T1 = milk with rennet (0.001 mL/100 mL of milk); T2 = milk with rennet (0.01 mL/100 mL of milk); and T3 = milk with rennet (0.1 mL/100 mL of milk). Bars not sharing common letters are significantly different (*p* < 0.05).

κ-CN breakdown rate % values at 12 and 24 h of incubation are presented in Figure 2. T1 showed the lowest κ-CN breakdown rates: 14.14 and 17.25% at 12 and 24 h, respectively. In contrast, T3 scored the highest κ-CN breakdown rate %, ranging from 20.62 to 45.14% at the same intervals. It has been noted that increasing rennet concentrations and incubation times led to a greater κ-CN breakdown [52,53].

### 3.3. Hydrodynamic Diameter and Surface Charge

The hydrodynamic diameter reflects the effective diameter of milk proteins as they move in a fluid, essentially measuring their size in a hydrated state. This parameter plays an important role in understanding the behavior and stability of milk proteins in different conditions [54]. The zeta potential of milk proteins, which indicates the electrical charge on their surfaces in a colloidal system, such as milk. This measurement is considered the key to estimating the stability and interactions of milk proteins in different cases [55].

Table 7 depicts that the different concentrations (treatments) and different incubation periods (time) at 4 °C and interaction between them (treatments × time) had a significant (*p* < 0.05) impact on both the hydrodynamic diameter and zeta potential of milk proteins in rennet-added SMS. The data on the hydrodynamic diameter and surface charge of milk proteins in SMS (control and rennet samples) during incubation at 4 °C are presented in Table 8.

In control milk (without rennet), the hydrodynamic diameter and zeta potential were around 18 µm and −4.10 mV, respectively, and different rennet concentrations in T1, T2, and T3 did not record any remarkable change in both the hydrodynamic diameter and zeta potential at 4 °C until 12 h of incubation, and these values were similar to the control. This could be attributed to weak enzyme activity because of the use of low rennet concentrations and low incubation temperatures (4 °C), which are unsuitable for enzyme activity. It is well known that the optimum temperature for rennet enzyme activity ranges from 35 to 40 °C [19,56]. However, increasing the incubation time to 24 h led to a significant increase (*p* < 0.05) in the hydrodynamic diameter at 24 h of incubation. These values ranged from 0.22 µm for T1 to 0.33 µm for T3. These results agreed with those obtained by others [57]. It was also noticed that the increased hydrodynamic diameter rate was positively correlated with the amount of rennet. This could be attributed to the role of a higher concentration of rennet in breaking down κ-CN (Table 6), which led to casein micelles losing their steric stabilization, decreasing their electrostatic repulsion, and becoming more prone to aggregation, clumping, and forming a more extensive protein network that has a larger hydrodynamic diameter than the casein micelles in normal milk [24].

Extending the incubation time to 24 h also negatively (*p* < 0.05) affected the zeta potential values of rennet-added SMS, especially with higher concentrations of rennet in (T3). This could be associated with the action of rennet in altering casein surface charge by breaking down κ-CN, which not only reduces the negative charge but also lowers the zeta potential. Similarly, the zeta potential of casein micelles decreases until the enzyme cleaves all the κ-casein during rennet action [58].

### 3.4. Rheological Properties

**Figure 3:**
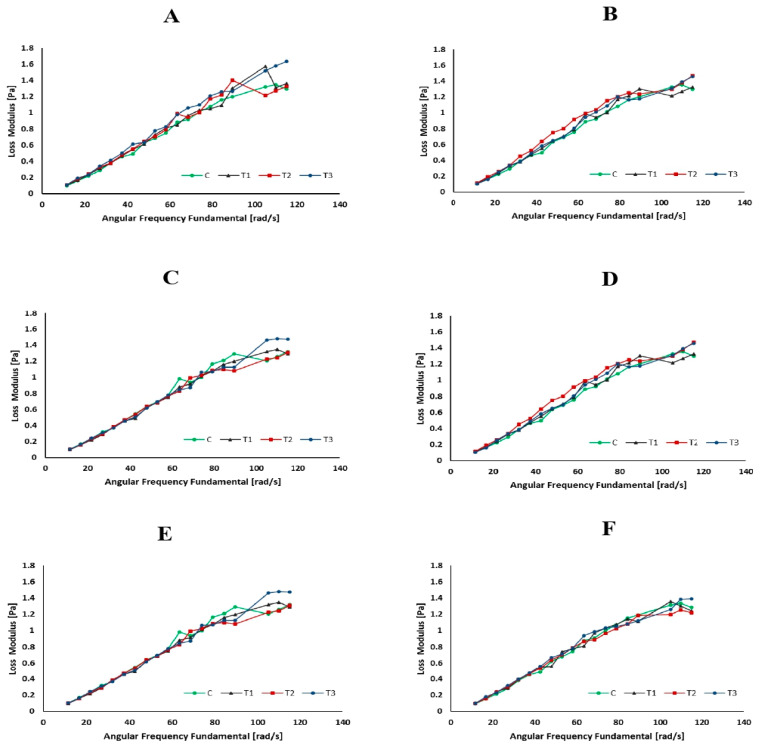
Loss modulus (Pa) of skim milk samples (control and rennet-added samples) at different incubation times ((**A**): 0 h, (**B**): 1 h, (**C**): 2 h, (**D**): 6 h, (**E**): 12 h, and (**F**): 24 h).

**Figure 4:**
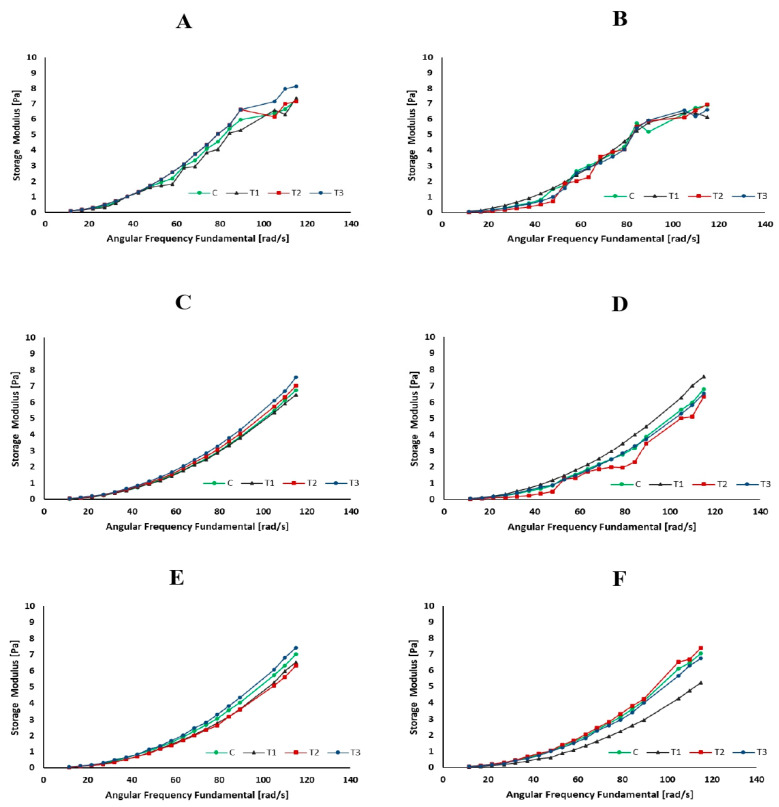
Storage modulus (Pa) of skim milk samples (control and rennet-added samples) at different incubation times ((**A**): 0 h, (**B**): 1 h, (**C**): 2 h, (**D**): 6 h, (**E**): 12 h, and (**F**): 24 h).

**Figure 5:**
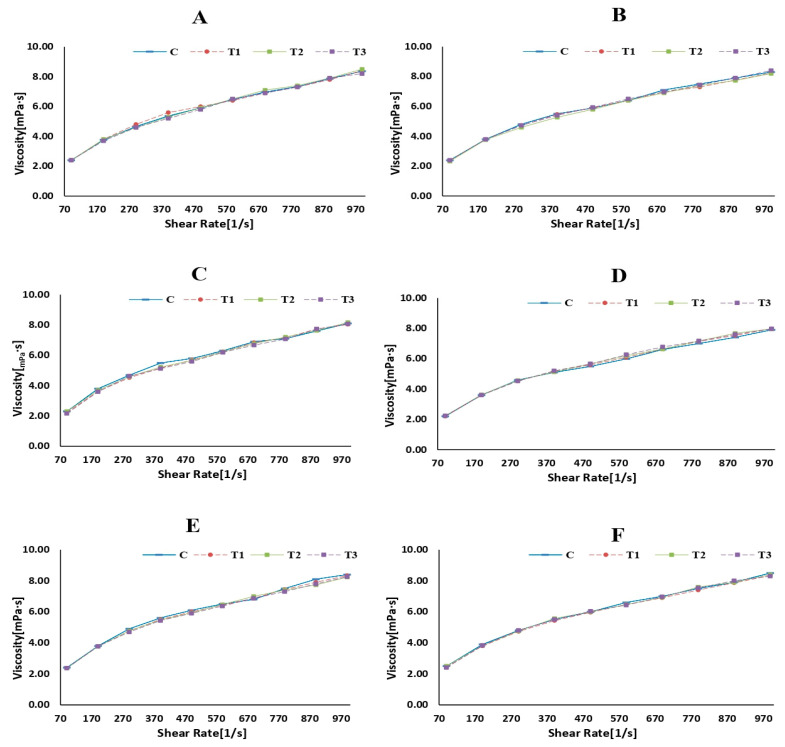
Viscosity (mPa·s) of skim milk samples (control and rennet-added samples) at different incubation times ((**A**): 0 h, (**B**): 1 h, (**C**): 2 h, (**D**): 6 h, (**E**): 12 h, and (**F**): 24 h).

Rheology is the scientific examination of how materials flow and deform. The storage modulus (G′), loss modulus (G″), and viscosity are commonly used parameters to describe the viscoelastic behavior of materials such as milk [59]. Figure 3, Figure 4 and Figure 5 represent the storage modulus (G′), loss modulus (G″), and viscosity (mPa·s) of SMS (control and rennet-added samples) during 24 h of incubation at 4 °C. The data obtained did not detect variations or distinct trends among all SMS samples at different incubation periods, indicating a fluctuation in the behavior of the samples throughout the various storage intervals. Although rennet resulted in gradual protein degradation over time (12 and 24 h), this degradation was not substantial enough to significantly change the rheological properties of the milk. Rennet significantly influences the rheological properties of milk, including its storage modulus (G′), loss modulus (G″), and viscosity [60]. The addition of rennet leads to the breakdown of κ-CN, resulting in changes in the viscoelastic properties of milk [61]. These protein changes tend to increase milk’s storage modulus (G′), suggesting a more structured or solidified state. Simultaneously, the loss modulus (G″) also changes and typically increases as the milk transitions from a predominantly liquid to a more gel-like state [62]. Rennet treatment also influences milk viscosity since the rennet enzymatic activity promotes protein aggregation and makes gel, increasing the milk’s viscosity.

### 3.5. Foam Properties

Milk foam is considered key to determining milk quality. Milk proteins (caseins and whey proteins) are the leading cause of foaming since they can reduce surface tension and stabilize air bubbles within milk [63]. In this context, the foamability (FA) of milk is defined as the proficiency of milk in making foam (capacity to form foam) during processing. On the other hand, foam stability (FS) reflects the maintenance of foam over time while keeping its structure without collapsing or converting to liquid [64].

**Figure 6:**
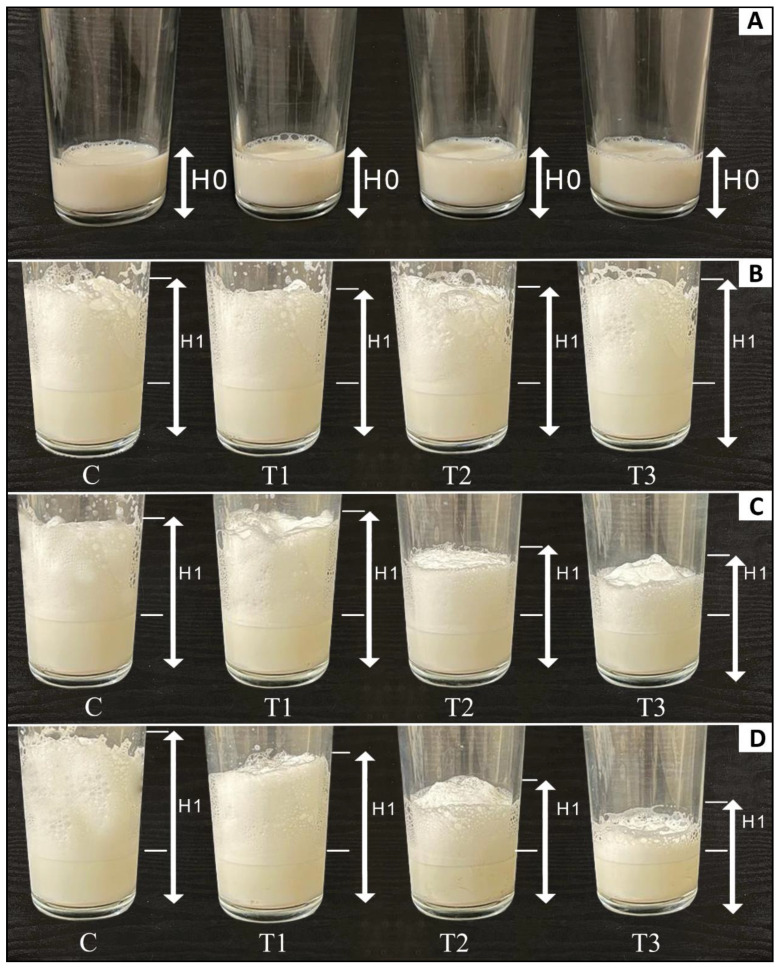
Foam ability of skim milk samples (control and rennet-added samples) at different incubation times. (**A**): H0, representing the initial height of the skim milk samples before conducting the foaming tests. (**B**–**D**): H1, indicating the height of skim milk samples after performing foaming tests at 0 h, 12, and 24 h of incubation time, respectively.

The results obtained in Table 9 showed that rennet amounts (treatments) and incubation periods (time), as well as the interaction between them (treatments × time), had a significantly (*p* < 0.05) negative impact on the foam ability and foam stability of SMS. The foam ability and foam stability of SMS (control and rennet-added samples) at 0, 12, and 24 h of incubation at 4 °C are illustrated in Table 10 and Figure 6. SMS (control and rennet-added samples) had similar values of FA and FS at zero hours. However, as the storage time advanced to 12 and 24 h, the increasing amount of rennet led to a significant (*p* < 0.05) decrease in FA and FS of rennet-added SMS, which reflects the impact of rennet action in breaking down casein proteins and reducing their surface-active role that is essential for foam formation and stabilization [65]. The casein molecules are important for their surface-active and foam-stabilizing properties [66]. When rennet’s enzymatic action alters the structure of the protein matrix, it weakens protein films around the air bubbles. This also explains the decrease in foam stability by increasing the addition of rennet [67]. Increasing the incubation time did not have a direct negative effect on FA and FS. However, the time-negative effect was associated with an increase in the breakdown of milk protein by rennet. Consequently, control samples (without rennet) maintained a high ability to produce foam during the various incubation stages.

### 3.6. Water-Holding Capacity

The water-holding capacity (WHC) of milk proteins refers to the ability of milk proteins (casein and whey proteins) to bind to water [68]. Casein micelles are responsible for trapping and stabilizing water molecules through hydrophilic interactions, contributing to milk’s viscosity [69]. Chymosin causes casein to coagulate and form a gel network. This coagulation significantly alters the WHC as the gel matrix formed effectively binds more water initially and expels some water during the formation of curds, especially after the gel is disturbed or cut. The results in Figure S1 indicate no WHC changes in SMS (control and rennet-added samples) during 24 h of incubation. This could be attributed to the fact that adding low levels of chymosin at low temperatures led to the breakdown of the κ-CN, but not to the extent that led to milk curd formation, and therefore, the WHC of SMS was not affected.

### 3.7. Oil Emulsifying Activity and Emulsion Stability

Milk protein emulsification is an important phenomenon involving stabilizing oil-in-water mixtures. These proteins (casein and whey proteins) have an amphipathic nature and possess both hydrophilic and hydrophobic domains [70]. These proteins reduce interfacial tension and form a protective layer around oil droplets, preventing coalescence and phase separation [71]. Emulsification enhances the physical stability and texture of dairy products. The emulsifying capacity of milk proteins is influenced by various factors, including protein concentration, pH, ionic strength, temperature, and enzyme treatments [12]. The emulsification properties are significantly influenced by the action of rennet [72]. Chymosin causes a reduction in the micellar steric repulsion and increases hydrophobic interactions, leading to the destabilization of the casein micelles [15]. As these micelles destabilize and aggregate, the emulsion’s stability is altered. Understanding the interplay between the rennet effect and protein emulsification is essential for optimizing dairy products’ texture, yield, and quality.

Table 9 indicates that rennet amounts (treatments) and incubation periods (time), as well as the interaction between them (treatments × time), had a significantly (*p* < 0.05) negative impact on the oil emulsifying activity (OEA) and emulsion stability (ES). The results obtained in Table 10 depict that OEA values were identical to the emulsion stability values of each sample, and the pasteurization and re-centrifuging processes did not affect the emulsion volume. All SMS (control and rennet-added samples) had similar OEA and ES values at 0 h. Increasing the incubation time to 12 h led to a significant (*p* < 0.05) increase in OEA and ES only in T3 (high levels of rennet). However, as the storage time advanced to 24 h, the increasing amount of rennet led to a significant (*p* < 0.05) increase in OEA and ES values. The observed increase in oil emulsion activity could be due to the action of chymosin in breaking down casein, which led to the unfolding of hydrophobic groups that were previously oriented toward the micelle interior [73]. The modified casein micelles may also become more interactive or be rearranged to stabilize the oil–water interface better, enhancing the emulsification. A change in emulsification properties after surface modification of casein micelles using transglutaminase enzymes has been reported [10].

## 4. Conclusions

The present study showed that low rennet concentrations under controlled low-temperature conditions (4 °C) can significantly modify the protein structure in skim milk, particularly affecting κ-casein, without resulting in aggregation or curd formation. The increase in NPN and NCN levels, besides the reduction in κ-casein in CGE data, confirmed the enzymatic action of rennet on milk proteins. However, rennet action did not significantly change the total protein, pH, or rheological properties of skim milk, which is beneficial for processing. By increasing the rennet concentration over time, the foamability and foam stability of the milk were negatively affected; however, the emulsifying activity and emulsion stability were enhanced. The experiment indicates that new, modified ingredients can be manufactured and used in products including fermented milks, yogurt, or products where altered milk protein functionality is needed. However, the tailored functionality will require the controlled enzymatic hydrolysis of milk proteins. To conclude, low concentrations of rennet at low storage temperatures reduced κ-casein, indicating casein modification, and milk proteins remained soluble. A more detailed study of the other functional properties and processing abilities of rennet milk needs to be explored. This study allows for further processing and the development of new dairy products.

## Figures and Tables

**Table 1:** Rennet amounts added to skim milk samples at 4 ± 1 °C.

Treatments	Rennet (mL/100 mL)
**C**	-
**T 1**	0.001
**T 2**	0.01
**T 3**	0.1

**Table 2:** Mean (*n* = 3) ± SE of the chemical composition of initial skim milk.

Fat%	Lactose%	TS%	SNF%	pH	TP%	NCN	NPN
0.18 ± 0.00	4.93 ± 0.01	9.72 ± 0.01	9.54 ± 0.00	6.62 ± 0.00	3.61 ± 0.00	0.67 ± 0.00	0.18 ± 0.00

SE: standard error. TS = total solids; SNF = solids not fat; TP = total protein; NCN = non-casein nitrogen; NPN = non-protein nitrogen.

**Table 3:** Mean squares and *p*-values (in parentheses) of pH, total protein, non-casein nitrogen, and non-protein nitrogen of skim milk samples during incubation time at 4 °C.

Factors	df	pH	TP	NCN	NPN
Treatments ^1^	3	0.03 (<0.05) *	0.001 (0.77)	0.52 (<0.05) *	0.16 (<0.05) *
Time ^2^ (h)	5	0.20 (<0.05) *	0.0007 (0.98)	0.53 (<0.05) *	0.14 (<0.05) *
Treatments ^1^ × Time ^2^ (h)	15	0.002 (0.70)	0.00007 (1)	0.06 (<0.05) *	0.01 (<0.05) *
Error	48	0.003	0.005	0.007	0.0004

* Significant at *p* < 0.05; TP = total protein; NCN = non-casein nitrogen; NPN = non-protein nitrogen. ^1^ Treatments: C = milk without rennet addition; T1 = milk with rennet (0.001 mL/100 mL of milk); T2 = milk with rennet (0.01 mL/100 mL of milk); and T3 = milk with rennet (0.1 mL/100 mL of milk). ^2^ Time = 0, 1, 2, 6, 12, and 24 h of incubation.

**Table 4:** Mean (*n* = 3) ± SE of chemical composition (*n* = 3) of skim milk samples after rennet addition during different incubation time periods at 4 °C.

			Time ^2^ (h)			
Treatments ^1^	0	1	2	6	12	24
			pH			
C	6.63 ^a^ ± 0.03	6.63 ^a^ ± 0.03	6.63 ^a^ ± 0.02	6.63 ^a^ ± 0.03	6.63 ^a^ ± 0.01	6.63 ^a^ ± 0.02
T1	6.63 ^a^ ± 0.03	6.63 ^a^ ± 0.03	6.56 ^ab^ ± 0.01	6.56 ^ab^ ± 0.02	6.56 ^ab^ ± 0.04	6.63 ^a^ ± 0.01
T2	6.55 ^ab^ ± 0.03	6.55 ^ab^ ± 0.03	6.55 ^ab^ ± 0.01	6.55 ^ab^ ± 0.02	6.55 ^ab^ ± 0.03	6.55 ^ab^ ± 0.01
T3	6.53 ^ab^ ± 0.03	6.46 ^b^ ± 0.02	6.53 ^ab^ ± 0.04	6.46 ^b^ ± 0.01	6.46 ^b^ ± 0.02	6.53 ^ab^ ± 0.04
			TP%			
C	3.58 ± 0.03	3.58 ± 0.03	3.58 ± 0.03	3.58 ± 0.03	3.58 ± 0.03	3.58 ± 0.03
T1	3.58 ± 0.03	3.60 ± 0.04	3.60 ± 0.04	3.60 ± 0.04	3.60 ± 0.04	3.60 ± 0.04
T2	3.58 ± 0.03	3.60 ± 0.04	3.60 ± 0.04	3.60 ± 0.04	3.60 ± 0.04	3.60 ± 0.04
T3	3.58 ± 0.03	3.60 ± 0.04	3.60 ± 0.04	3.60 ± 0.04	3.60 ± 0.04	3.60 ± 0.04
			NCN%			
C	0.67 ^g^ ± 0.02	0.67 ^g^ ± 0.02	0.67 ^g^ ± 0.02	0.67 ^g^ ± 0.02	0.67 ^g^ ± 0.02	0.67 ^g^ ± 0.02
T1	0.67 ^g^ ± 0.02	0.73 ^fg^ ± 0.02	0.77 ^fg^ ± 0.03	0.99 ^de^ ± 0.07	1.11 ^cd^ ± 0.07	1.26 ^bc^ ± 0.06
T2	0.67 ^g^ ± 0.02	0.78 ^fg^ ± 0.02	0.80 ^fg^ ± 0.01	1.13 ^c^ ± 0.08	1.25 ^bc^ ± 0.07	1.34 ^ab^ ± 0.06
T3	0.67 ^g^ ± 0.02	0.79 ^fg^ ± 0.01	0.88 ^ef^ ± 0.02	1.19 ^bc^ ± 0.08	1.31 ^b^ ± 0.06	1.48 ^a^ ± 0.07
			NPN%			
C	0.18 ^i^ ± 0.01	0.18 ^i^ ± 0.01	0.18 ^i^ ± 0.01	0.18 ^i^ ± 0.01	0.18 ^i^ ± 0.01	0.18 ^i^ ± 0.01
T1	0.18 ^i^ ± 0.01	0.21 ^hi^ ± 0.01	0.24 ^g^ ± 0.00	0.31 ^f^ ± 0.00	0.39 ^e^ ± 0.00	0.43 ^d^ ± 0.00
T2	0.18 ^i^ ± 0.01	0.24 ^gh^ ± 0.00	0.26 ^g^ ± 0.00	0.36 ^e^ ± 0.00	0.47 ^c^ ± 0.01	0.54 ^b^ ± 0.01
T3	0.18 ^i^ ± 0.01	0.26 ^g^ ± 0.00	0.31 ^f^ ± 0.01	0.43 ^d^ ± 0.01	0.54 ^b^ ± 0.00	0.68 ^a^ ± 0.00

SE: standard error; TP = total protein; NCN = non-casein nitrogen; NPN = non-protein nitrogen. ^a–i^ Means in columns and rows (treatments × time) not sharing a common superscript are different at *p* < 0.05. ^1^ Treatments: C = milk without rennet addition; T1 = milk with rennet (0.001 mL/100 mL of milk); T2 = milk with rennet (0.01 mL/100 mL of milk); and T3 = milk with rennet (0.1 mL/100 mL of milk). ^2^ Time = 0, 1, 2, 6, 12, and 24 h of incubation at 4 °C.

**Table 5:** Mean squares and *p*-values (in parentheses) relative protein fractions measured using capillary gel electrophoresis of skim milk samples during incubation at 4 °C.

Factors	df	LMWP	α-LA	β-LG	γ-CN	β-Casein	α-S1	α-S2	κ-CN	HMWP
Treatments ^1^	3	1.70 (<0.05) *	0.48 (0.25)	1.18 (0.34)	0.16 (0.005) *	9.41 (<0.05) *	1.65 (0.30)	7.14 (0.04)	2.46 (<0.05) *	0.09 (<0.05) *
Time ^2^ (h)	5	9.42 (<0.05) *	2.32 (<0.05) *	3.59 (<0.05) *	1.80 (<0.05) *	6.40 (0.08)	8.39 (<0.05) *	10.98 (0.003)	9.97 (<0.05) *	0.17 (<0.05) *
Treatments ^1^ × Time ^2^ (h)	15	1.05 (<0.05) *	0.25 (0.72)	0.65 (0.83)	0.23 (<0.05) *	1.42 (0.95)	2.74 (0.03)	1.88 (0.73)	1.45 (<0.05) *	0.02 (<0.05) *
Error	48	0.05	0.34	1.03	0.04	3.12	1.32	2.53		0.005

* Significant at *p* < 0.05; LMWP = low-molecular-weight peptides; HMWP = high-molecular-weight peptides; ^1^ treatments: C = milk without rennet addition; T1 = milk with rennet (0.001 mL/100 mL of milk); T2 = milk with rennet (0.01 mL/100 mL of milk); and T3 = milk with rennet (0.1 mL/100 mL of milk). ^2^ Time = 0, 1, 2, 6, 12, and 24 h of incubation at 4 °C.

**Table 6:** Mean (*n* = 3) ± SE of relative protein fractions measured using capillary gel electrophoresis of skim milk samples after rennet addition during different incubation periods at 4 °C.

Treatments ^1^	Time ^2^	LMWP	α-LA	β-LG	γ-CN	β-Casein	α-S1	α-S2	κ-CN	HMWP
C	0	2.31 ^fg^ ± 0.12	4.69 ± 0.46	10.98 ± 0.34	1.38 ^efg^ ± 0.07	29.13 ± 0.72	34.75 ± 0.78	9.10 ± 1.24	7.60 ^abc^ ± 0.39	0.06 ^g^ ± 0.03
	1	2.14 ^g^ ± 0.11	3.57 ± 0.37	11.73 ± 0.33	1.32 ^efg^ ± 0.20	31.15 ± 0.68	32.62 ^abc^ ± 0.61	8.99 ± 1.16	8.37 ^a^ ± 0.24	0.12 ^efg^ ± 0.02
	2	2.58 ^fg^ ± 0.15	4.70 ± 0.17	10.72 ± 0.23	1.37 ^efg^ ± 0.06	29.80 ± 0.95	34.08 ^abc^ ± 0.46	9.10 ± 0.31	7.62 ^abc^ ± 0.27	0.03 ^g^ ± 0.02
	6	2.77 ^f^ ± 0.10	4.56 ± 0.26	11.11 ± 0.25	1.31 ^efg^ ± 0.09	29.03 ± 0.97	34.08 ^abc^ ± 0.23	9.14 ± 0.22	7.97 ^a^ ± 0.28	0.02 ^g^ ± 0.01
	12	2.89 ^f^ ± 0.02	4.68 ± 0.30	10.73 ± 0.64	1.32 ^efg^ ± 0.05	28.24 ± 1.72	35.43 ^ab^ ± 0.22	8.74 ± 0.51	7.90 ^ab^ ± 0.29	0.08 ^g^ ± 0.02
	24	2.51 ^fg^ ± 0.34	5.01 ± 0.43	11.12 ± 0.26	1.51 ^efg^ ± 0.12	30.18 ± 0.28	32.71 ^abc^ ± 1.51	9.29 ± 1.28	7.52 ^abc^ ± 0.53	0.14 ^efg^ ± 0.02
T1	0	2.80 f ± 0.09	4.46 ± 0.60	9.99 ± 1.05	1.05 ^gh^ ± 0.21	27.87 ± 1.14	34.28 ^ab^c ± 0.99	11.83 ± 2.01	7.59 ^abc^ ± 0.17	0.13 ^efg^ ± 0.02
	1	2.10 g ± 0.34	3.83 ± 0.30	11.05 ± 0.14	1.39 ^efg^ ± 0.10	29.34 ± 0.46	33.24 ^abc^ ± 0.63	11.34 ± 0.13	7.57 ^abc^ ± 0.72	0.14 ^efg^ ± 0.05
	2	2.51 ^fg^ ± 0.03	4.67 ± 0.58	11.60 ± 0.81	1.38 ^efg^ ± 0.07	27.60 ± 0.62	33.89 ^ab^ ± 0.21	9.74 ± 0.87	8.54 ^a^ ± 0.29	0.07 ^g^ ± 0.01
	6	2.82 ^f^ ±0.05	5.06 ± 0.15	11.30 ± 0.66	1.49 ^efg^ ± 0.04	27.73 ± 1.19	33.60 ^abc^ ± 0.60	9.51 ± 0.14	8.47 ^a^ ± 0.16	0.03 ^g^ ± 0.03
	12	3.66 ^e^ ± 0.07	4.98 ± 0.19	10.54 ± 0.67	1.71 ^def^ ± 0.03	28.73 ± 1.14	34.77 ^abc^ ± 0.18	8.82 ± 0.25	6.62 ^bcd^ ± 0.01	0.18 ^defg^ ± 0.03
	24	4.57 ^c^ ± 0.07	4.73 ± 0.55	10.02 ± 1.00	2.18 ^bc^ ± 0.09	27.11 ± 1.17	33.22 ^abc^ ± 0.89	11.75 ± 1.93	6.11 ^de^ ± 0.08	0.32 ^bcd^ ± 0.13
T2	0	2.81 ^f^ ± 0.08	4.74 ± 0.38	10.40 ± 0.62	1.15 ^gh^ ± 0.13	28.39 ± 0.60	31.89 ^bc^ ± 0.63	11.97 ± 0.82	8.34 ^a^ ± 0.18	0.31 ^bcde^ ± 0.02
	1	2.27 ^fg^ ± 0.16	4.55 ± 0.25	11.55 ± 0.24	1.31 ^efg^ ± 0.12	29.36 ± 0.53	31.98 ^abc^ ± 0.54	10.64 ± 0.78	8.11 ^a^ ± 0.11	0.21 ^defg^ ± 0.05
	2	2.16 ^g^ ± 0.17	4.69 ± 0.07	11.07 ± 0.46	1.48 ^efg^ ± 0.08	27.92 ± 0.29	35.04 ^abc^ ± 0.72	9.73 ± 0.34	7.75 ^ab^ ± 0.30	0.15 ^efg^ ± 0.02
	6	2.65 ^fg^ ± 0.04	5.02 ± 0.31	10.93 ± 0.21	1.37 ^efg^ ± 0.04	28.14 ± 1.06	35.49 ^a^ ± 0.35	8.77 ± 0.12	7.59 ^abc^ ± 0.15	0.03 ^g^ ± 0.04
	12	3.81 ^de^ ± 0.03	5.07 ± 0.25	10.25 ± 0.31	1.83 ^cde^ ± 0.07	29.53 ± 0.66	34.68 ^abc^ ± 0.76	8.15 ± 0.10	6.38 ^cde^ ± 0.03	0.29 ^cdef^ ± 0.02
	24	5.09 ^b^ ± 0.07	4.99 ± 0.32	10.51 ± 0.63	2.40 ^ab^ ± 0.06	27.89 ± 0.65	31.26 ^c^ ± 0.57	12.02 ± 0.76	5.39 ^e^ ± 0.09	0.45 ^ab^ ± 0.04
T3	0	2.50 ^fg^ ± 0.13	4.38 ± 0.04	9.38 ± 0.59	1.04 ^gh^ ± 0.11	29.59 ± 1.16	34.44 ^abc^ ± 0.37	11.10 ± 1.47	7.50 ^abc^ ± 0.29	0.07 ^fg^ ± 0.02
	1	2.04 ^g^ ± 0.02	3.24 ± 0.05	11.78 ± 1.11	0.82 ^h^ ± 0.15	31.35 ± 0.84	33.99 ^abc^ ± 0.83	8.37 ± 0.40	8.29 ^a^ ± 0.20	0.12 ^g^ ± 0.02
	2	2.11 ^g^ ± 0.09	4.31 ± 0.17	10.55 ± 0.35	1.17 ^fgh^ ± 0.18	29.64 ± 1.16	35.21 ^ab^ ± 0.20	9.68 ± 0.16	7.27 ^abc^ ± 0.02	0.06 ^g^ ± 0.02
	6	2.77 ^f^ ± 0.07	5.25 ± 0.41	11.29 ± 0.58	1.35 ^efg^ ± 0.08	27.87 ± 1.68	33.53 ^abc^ ± 0.63	9.59 ± 0.55	8.31 ^a^ ± 0.34	0.03 ^g^ ± 0.10
	12	4.13 ^d^ ± 0.11	5.20 ± 0.37	10.38 ± 0.40	1.94 ^cd^ ± 0.05	29.19 ± 1.58	33.90 ^abc^ ± 0.42	8.75 ± 0.39	6.12 ^de^ ± 0.03	0.39 ^abc^ ± 0.01
	24	5.69 ^a^ ± 0.09	4.43 ± 0.08	9.26 ± 0.58	2.65 ^a^ ± 0.13	28.83 ± 1.26	33.50 ^abc^ ± 0.20	10.89 ± 1.40	4.23 ^f^ ± 0.11	0.53 ^a^ ± 0.07

SE: standard error; LMWP = low-molecular-weight peptides; HMWP = high-molecular-weight peptides; ^a–h^ means in columns and rows (treatments × time) not sharing a common superscript are different at *p* < 0.05. ^1^ Treatments: C = milk without rennet addition; T1 = milk with rennet (0.001 mL/100 mL of milk); T2 = milk with rennet (0.01 mL/100 mL of milk); and T3 = milk with rennet (0.1 mL/100 mL of milk). ^2^ Time = 0, 1, 2, 6, 12, and 24 h of incubation at 4 °C.

**Table 7:** Mean squares and *p*-values (in parentheses) of hydrodynamic diameter (µm) and zeta potential (mv) of protein in skim milk samples during incubation at 4 °C.

Factors	df	Hydrodynamic Diameter	Zeta Potential
Treatments ^1^	3	0.003 (<0.05)	0.00001 (<0.05)
Time ^2^ (h)	5	0.007 (<0.05)	0.00005 (<0.05)
Treatments ^1^ × Time ^2^ (h)	15	0.002 (<0.05)	0.00003 (<0.05)
Error	48	0.00001	0.0000003

^1^ Treatments: C = milk without rennet addition; T1 = milk with rennet (0.001 mL/100 mL of milk); T2 = milk with rennet (0.01 mL/100 mL of milk); and T3 = milk with rennet (0.1 mL/100 mL of milk). ^2^ Time = 0, 1, 2, 6, 12, and 24 h of incubation.

**Table 8:** Mean (*n* = 3) ± SE of hydrodynamic diameter (µm) and zeta potential (mv) of protein in skim milk samples after the rennet addition for different incubation time periods at 4 °C.

			Time ^2^ (h)			
Treatments ^1^	0	1	2	6	12	24
		Hydrodynamic Diameter (µm)				
C	0.18 ^efg^ ± 0.00	0.19 ^defg ±^ 0.00	0.18 ^def^ ± 0.00	0.18 ^g^ ± 0.00	0.18 ^fg^ ± 0.00	0.18 ^efg ±^ 0.00
T1	0.18 ^efg^ ± 0.00	0.18 ^defg^ ± 0.00	0.19 ^de^ ± 0.00	0.18 ^efg^ ± 0.00	0.18 ^efg^ ± 0.00	0.22 ^c^ ± 0.00
T2	0.18 ^defg^ ± 0.00	0.19 ^defg^ ± 0.00	0.18 ^defg^ ± 0.00	0.18 ^efg^ ± 0.00	0.19 ^def^ ± 0.00	0.24 ^b^ ± 0.00
T3	0.18 ^defg^ ± 0.00	0.18 ^defg^ ± 0.00	0.19 ^d^ ± 0.00	0.18 ^efg^ ± 0.00	0.18 ^defg^ ± 0.00	0.33 ^a^ ± 0.00
		Zeta potential (mv)				
C	−4.10 ^ab^ ± 0.53	−4.83 ^abc^ ± 0.27	−4.86 ^abc^ ± 0.15	−5.69 ^bc^ ± 0.23	−5.58 ^abc^ ± 0.21	−5.41 ^abc^ ± 0.29
T1	−5.10 ^abc^ ± 0.25	−5.31 ^abc^ ± 0.17	−5.06 ^abc^ ± 0.39	−5.34 ^abc^ ± 0.12	−5.15 ^abc^ ± 0.09	−5.19 ^abc^ ± 0.52
T2	−5.40 ^abc^ ± 0.27	−4.39 ^ab^ ± 0.42	−5.12 ^abc^ ± 0.32	−5.58 ^abc^ ± 0.07	−5.18 ^abc^ ± 0.41	−6.03 ^c^ ± 0.19
T3	−5.22 ^abc^ ± 0.36	−4.01 ^a^ ± 0.34	−4.95 ^abc^ ± 0.07	−4.52 ^abc^ ± 0.28	−5.01 ^abc^ ± 0.29	−22.67 ^d^ ± 0.33

SE: standard error; ^a–g^ Means in columns and rows (treatments × time) not sharing a common superscript are different at *p*< 0.05. ^1^ Treatments: C = milk without rennet addition; T1 = milk with rennet (0.001 mL/100 mL of milk); T2 = milk with rennet (0.01 mL/100 mL of milk); and T3 = milk with rennet (0. 1 mL/100 mL of milk). ^2^ Time = 0, 1, 2, 6, 12, and 24 h of incubation at 4 °C.

**Table 9:** Mean squares and *p*-values (in parentheses) of foam ability %, foam stability %, oil emulsifying activity %, and emulsion stability% of protein in skim milk samples during incubation at 4 °C.

Factors	df	Foam Ability %	Foam Stability %	Oil Emulsifying Activity %	Emulsion Stability %
Treatments ^1^	3	6809 (<0.05) *	1414 (<0.05) *	81.52 (<0.05) *	81.52 (<0.05) *
Time ^2^ (h)	2	14,368 (<0.05) *	1682 (<0.05) *	226.03 (<0.05) *	226.03 (<0.05) *
Treatments ^1^ × Time ^2^ (h)	6	1952 (<0.05) *	366 (<0.05) *	44.77 (<0.05) *	44.77 (<0.05) *
Error	24	103	28.88	0.81	0.81

* Significant at *p* < 0.05. ^1^ Treatments: C = milk without rennet addition; T1 = milk with rennet (0.001 mL/100 mL of milk); T2 = milk with rennet (0.01 mL/100 mL of milk); and T3 = milk with rennet (0.1 mL/100 mL of milk). ^2^ Time = 0, 1, 2, 6, 12, and 24 h of incubation.

**Table 10:** Mean (*n* = 3) ± SE of foam ability %, foam stability %, oil emulsifying activity %, and emulsion stability % of protein in skim milk samples after the addition of rennet during different incubation time periods at 4 °C.

		Time (h)	
Treatments ^1^	0	12	24
		Foam Ability %	
C	211.67 ^a^ ± 12.44	203.33 ^ab^ ± 6.44	203.00 ^ab^ ± 4.93
T1	197.33 ^ab^ ± 3.48	181.67 ^b^c ± 1.85	139.33 ^d^ ± 2.84
T2	208.00 ^a^ ± 9.45	168.33 ^c^ ± 1.85	119.67 ^d^ ± 2.33
T3	207.33 ^a^ ± 7.17	124.67 ^d^ ± 2.96	85.67 ^e^ ± 3.28
		Foam stability %	
C	68.67 ^a^ ± 2.18	71.67 ^a^ ± 6.20	72.33 ^a^ ± 3.17
T1	73.33 ^a^ ± 5.48	60.33 ^ab^ ± 1.76	53.67 ^bc^ ± 1.20
T2	67.67 ^a^ ± 1.76	47.33 ^cd^ ± 2.03	39.33 ^de^ ± 0.88
T3	69.33 ^a^ ± 3.92	36.33 ^e^ ± 0.88	21.00 ^f^ ± 1.73
		Oil emulsifying activity %	
C	30.00 ^a^ ± 0.58	30.00 ^a^ ± 0.33	30.00 ^a^ ± 0.00
T1	30.00 ^a^ ± 0.67	30.00 ^a^ ± 0.58	35.00 ^c^ ± 0.00
T2	30.00 ^a^ ± 0.58	30.00 ^a^ ± 0.00	42.00 ^b^ ± 0.00
T3	30.00 ^a^ ± 1.16	35.00 ^c^ ± 0.00	46.00 ^a^ ± 0.57
		Emulsion stability %	
C	30.00 ^a^ ± 0.58	30.00 ^a^ ± 0.33	30.00 ^a^ ± 0.00
T1	30.00 ^a^ ± 0.67	30.00 ^a^ ± 0.58	35.00 ^c^ ± 0.00
T2	30.00 ^a^ ± 0.58	30.00 ^a^ ± 0.00	42.00 ^b^ ± 0.00
T3	30.00 ^a^ ± 1.16	35.00 ^c^ ± 0.00	46.00 ^a^ ± 0.57

SE: standard error. ^a–f^ Means in columns and rows (treatments × time) not sharing a common superscript are different at *p* < 0.05. ^1^ Treatments: C = milk without rennet addition; T1 = milk with rennet (0.001 mL/100 mL of milk); T2 = milk with rennet (0.01 mL/100 mL of milk); and T3 = milk with rennet (0.1 mL/100 mL of milk).

## Data Availability

Data is contained within the article or Supplementary Material.

## Supplementary Materials

The following supporting information can be downloaded at: https://www.mdpi.com/article/10.3390/foods13030447/s1, Figure S1: No changes in the water holding capacity of SMS (control and rennet-added samples) after centrifuging at 24 h incubation.
