# Supplementary material for: Changes in Milk Protein Functionality at Low Temperatures and Rennet Concentrations
Source: Foods. 2024 Jan 30;13(3):447. doi: 10.3390/foods13030447 (PMC10855877; doi:10.3390/foods13030447)
Supplement: Supplementary file 1 [file foods-13-00447-s001.zip › foods-2839548-supplementary.pdf]

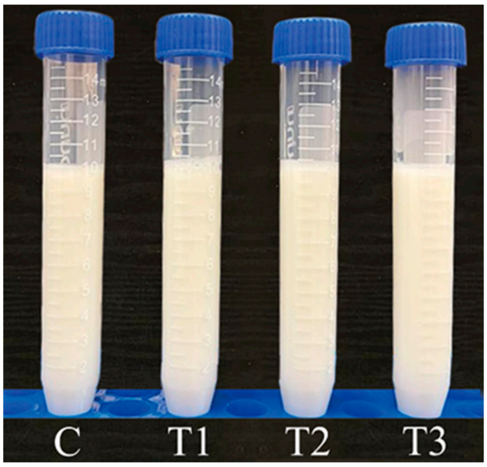


**Figure S1**. No changes in the water holding capacity of SMS (control and rennet-added samples) after centrifuging at 24 h incubation
